# Serum Uric Acid as a Putative Biomarker in Prodromal Parkinson’s Disease: Longitudinal Data from the PPMI Study^1^

**DOI:** 10.3233/JPD-230007

**Published:** 2023-07-25

**Authors:** Christos Koros, Athina-Maria Simitsi, Nikolaos Papagiannakis, Anastasia Bougea, Andreas Prentakis, Dimitra Papadimitriou, Ioanna Pachi, Ion Beratis, Evangelia Stanitsa, Efthalia Angelopoulou, Roubina Antonelou, Marianna Bregianni, Konstantinos Lourentzos, Sokratis G. Papageorgiou, Anastasios Bonakis, Xenia Geronicola Trapali, Maria Stamelou, Leonidas Stefanis

**Affiliations:** a 1^st^ Department of Neurology, Eginition Hospital, National and Kapodistrian University of Athens, Athens, Greece; b Nuclear Medicine Unit, Attikon Hospital, Athens, Greece; c Neurology Clinic, Henry Dunan Hospital, Athens, Greece; d 2^nd^ Department of Neurology, Attikon Hospital, National and Kapodistrian University of Athens, Athens, Greece; e Neurology Clinic, Philipps University, Marburg, Germany; f Parkinsons disease and Movement Disorders Dept., HYGEIA Hospital, Athens, Greece

**Keywords:** Prodromal Parkinson’s disease, uric acid, REM sleep behavior disorder, hyposmia

## Abstract

**Background:**

The role of blood uric acid as a biomarker in symptomatic motor PD has been increasingly established in the literature.

**Objective:**

Our present study assessed the role of serum uric acid as a putative biomarker in a prodromal PD cohort [REM Sleep Behavior disorder (RBD) and Hyposmia] followed longitudinally.

**Methods:**

Longitudinal 5-year serum uric acid measurement data of 39 RBD patients and 26 Hyposmia patients with an abnormal DATSCAN imaging were downloaded from the Parkinson’s Progression Markers Initiative database. These cohorts were compared with 423 de novo PD patients and 196 healthy controls enrolled in the same study.

**Results:**

After adjusting for age, sex, body mass index, and concomitant disorders (hypertension/gout), baseline and longitudinal serum uric acid levels were higher in the RBD subgroup as compared to the established PD cohort (*p* = 0.004 and *p* = 0.001). (Baseline RBD 6.07±1.6 vs. Baseline PD 5.35±1.3 mg/dL and Year-5 RBD 5.7±1.3 vs. Year-5 PD 5.26±1.33). This was also true for longitudinal measurements in the Hyposmic subgroup (*p* = 0.008) (Baseline Hyposmic 5.7±1.6 vs. PD 5.35±1.3 mg/dL and Year-5 Hyposmic 5.58±1.6 vs. PD 5.26±1.33).

**Conclusion:**

Our results indicate that serum uric acid levels are higher in prodromal PD subjects with ongoing dopaminergic degeneration compared to those with manifest PD. These data indicate that the well-established decrease in the levels of serum uric acid occurs with the transition from prodromal to clinical PD. Whether the higher levels of serum uric acid observed in prodromal PD may provide protection against conversion to full-blown clinical PD will require further study.

## INTRODUCTION

Prodromal Parkinson’s disease (PD) corresponds to the premotor phase of PD lasting many years. During this phase the neurodegeneration process is active and ongoing and typical prodromal non-motor symptoms like REM sleep behavior disorder (RBD), depression, hyposmia, and constipation emerge. The progression to the non-motor and motor manifestations of PD aligns well with the neuropathological staging system proposed by Braak [1]. Numerous risk factors have been thoroughly described in previous studies and they facilitate the assessment of the probability to develop motor PD [2]. Such markers have been incorporated in the recent MDS criteria for prodromal PD [3].

Oxidative stress is thought to play a vital role in the pathogenesis of PD [4]. An endogenous antioxidant molecule, uric acid, is considered to be a buffering agent and to possibly protect dopaminergic neurons from oxidative stress in PD. Uric acid exerts its antioxidant function mainly by means of ferrum chelation, although other mechanisms may also contribute to this effect. *In vitro* studies have shown that uric acid could induce autophagy activation and ameliorate alpha-synuclein (SNCA) accumulation [5]. In an *in vivo* PD model, uric acid demonstrated neuroprotective properties for dopaminergic neurons by means of increased expression of nuclear factor E2-related factor 2 (Nrf2) and three Nrf2-responsive genes [6]. In certain PD population studies, PD patients exhibited lower serum uric level in comparison to healthy controls [7, 8] but in other cohorts this difference was less evident [9].

The Parkinson’s Progression Markers Initiative (PPMI) study evaluated the 5-year change of clinical, imaging, and biochemical parameters in de novo PD patients, in subjects with prodromal PD symptoms (RBD and hyposmia) and in healthy controls. The aim of our present study is to determine whether there are baseline and longitudinal differences in serum uric acid levels between Prodromal individuals (RBD and Hyposmic) and PD patients or healthy controls enrolled in the PPMI study, so as to ascertain the possible relationship of this emerging biomarker to the evolution of PD at its earlier stages, before the emergence of the classical clinical signs.

## MATERIALS AND METHODS

Data used in the preparation of this article were obtained on 04/18/2020 (03/27/2023 last update) from the PPMI database (http://www.ppmi-info.org/access-data-specimens/download-data), RRID:SCR_006431. For up-to-date information on the study, visit http://www.ppmi-info.org. The present study was conducted in agreement with the principles of the Declaration of Helsinki. Signed informed consent was obtained from all participants recruited. The study was approved by the Scientific Board of all PPMI sites involved (including the Scientific Board of Attikon and Eginition hospitals). Longitudinal 5-year serum uric acid measurement data of 39 RBD participants and 26 Hyposmia participants were downloaded from the PPMI database. Moreover, we obtained data from 423 de novo sporadic PD patients and 196 healthy controls from the same database.

The Prodromal cohort in the PPMI study included premotor subjects with prodromal PD symptoms (RBD or hyposmia/reduced olfaction) [10, 11]. RBD was diagnosed based on clinical history and sleep related scales along with polysomnography, if available. 34 out of 39 RBD participants in our PPMI subgroup were PSG-confirmed. The remaining 5 were “probable RBD” based on the medical history and the RBD-questionnaire but the number of this group appears too small in order to have any meaningful comparisons if we consider separately the 5 “probable RBD” vs. the 34 PSG-confirmed RBD participants. In the hyposmia cohort, olfaction was measured using the University of Pennsylvania Smell Identification Test (UPSIT). Hyposmia was defined as a score of < 10th percentile for age and sex. PPMI inclusion criteria enriched the RBD and hyposmia cohorts with individuals with dopamine transporter binding deficit. Most participants with a normal DATSCAN were excluded from the study with the exception of 10% of those without a DAT binding deficit with the goal of keeping site investigators blinded to DAT SPECT results. A total number of 65 Prodromal subjects (RBD, *n* = 39 and Hyposmic, *n* = 26) were finally enrolled. An additional group of 57 RBD and 93 Hyposmic individuals with a normal DATSCAN during the screening visit were excluded from follow up in PPMI (however serum uric acid measurements from samples of this cohort collected during the screening visit were also available in the database of thestudy).

Biochemical analyses (including measurements of uric acid level in serum) have been carried out in Covance laboratories in a uniform fashion, as per the study protocol.

Statistical analysis for baseline comparisons between the prodromal subjects (RBD and Hyposmic subgroups) and the idiopathic PD cohort/healthy controls was performed using univariate ANCOVA. Factors that could have an impact on uric acid [age/sex/body mass index (BMI)/concomitant disorders and medication (hypertension and gout)] were used as covariates in the analysis. Repeated measures ANCOVA (tests of within- and between- subjects effects) was used to examine the 5-year longitudinal changes of uric acid level (RBD and Hyposmic subgroups vs. sporadic PD and healthy controls). Time, group, or combined Time*Group interactions were assessed using assessment of within-subjects effects. Statistical significance was set at *p* < 0.05. Additionally, a nested ANCOVA model, with conversion subgroups nested inside RBD and Hyposmia subgroups, was used to estimate the differences between patients converted or not.

Pearson or Spearman correlations (according to the normality of group values) were calculated between uric acid level, duration since initiation of RBD and various baseline parameters (MDS-UPDRS score part III, 2-year and 4-year change in MDS UPDRS III and Montreal Cognitive Assessment (MoCA) score) in the RBD cohort and the hyposmia cohort.

Finally, in order to assess the putative impact of uric acid levels on clinical progression of patients in the prodromal cohort and determine if high and low uric acid groups follow a different prospective clinical outcome, we compared high serum uric acid level- and low serum uric acid level- subgroups (at screening) in terms of motor deterioration (MDS-UPDRS III score at baseline, year 2, year 4 and the 2-year and 4-year score change) using univariate ANCOVA with age, sex, and BMI as covariates. We defined cut-off as the median of baseline uric acid level 5.9 md/dL for the RBD group and 5.75 for the Hyposmia group (as a result high serum uric acid group was that with measurements above the median and low serum acid group below the median respectively). The statistical analyses were performed using commercially available software (SPSS, Version 20.0).

## RESULTS

**Fig. 1:**
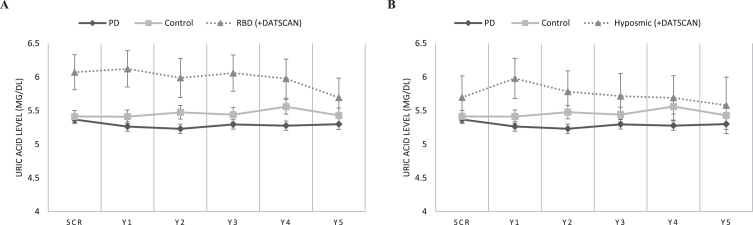
Longitudinal measurements of serum uric acid levels in: A) the RBD cohort vs. idiopathic PD and healthy controls, and B) the Hyposmic cohort vs. idiopathic PD and healthy controls. Statistical analysis has been performed using repeated measures linear ANCOVA (with age, sex, BMI, and presence of hypertension/gout as covariates). PPMI timepoints of visits SCR, V04, V06, V08, V10 and V12 correspond to Years 0, 1, 2, 3, 4, and 5, respectively. Error bars represent Standard Error of Mean.

After adjusting for age, sex, BMI, and the presence of hypertension and/or gout, baseline and 5-year longitudinal serum uric acid measurements were higher in the RBD cohort as compared to the PD cohort (*p* = 0.004 and *p* = 0.001, respectively). [Baseline RBD subgroup uric acid level was 6.07 mg/dL (95% CI 5.57 to 6.57) vs. Baseline PD 5.35 mg/dL (95% CI 5.23 to 5.47)]. There was no significant effect of Time*Group interaction on uric acid level (Within subject effects, *p* = 0.752). Baseline and longitudinal serum uric acid measurements did not differ statistically between RBD subjects and healthy controls (despite a trend for higher uric acid in the RBD group) (Between subjects effects, *p* = 0.145 and *p* = 0.166). [Baseline RBD subgroup uric acid level was 6.07 mg/dL (95% CI 5.57 to 6.57) vs. Baseline HC 5.41 mg/dL(95% CI 5.23 to 5.59)]. No significant effect of Time*Group interaction on uric acid level was evidenced (Within subject effects, *p* = 0.087) (Fig. 1A, Table 1).

**Table 1:** Baseline demographic, motor, and non-motor characteristics in RBD prodromal, Hyposmic prodromal idiopathic PD and healthy controls (Mean ± SD)

Feature	RBD cohort	Hyposmia cohort	Idiopathic PD	Healthy Controls
	(N = 39)	(N = 26)	(N = 421)	(N = 195)
*Demographics*				
	mean ± SD	mean ± SD	mean ± SD	mean ± SD
Age (y)	69.6(±5.5)	68.1(±6.2)	61.63(±9.69)	60.77 (±11.26)
	frequencies	frequencies	frequencies	Frequencies
Sex (male / female)	33(85%) / 16 (15%)	18(69%) / 8 (31%)	277(65.8%) / 144(34.2%)	125 (64.1%) / 70 (35.9%)
Hypertension History/Medication	20.5% (8/39)	50% (13/26)	37.5% (158/421)	40.5% (79/195)
Gout History/Medication	7.7% (3/39)	3.9% (1/26)	1.4% (6/421)	4.1% (8/195)
	mean ± SD	mean ± SD	mean ± SD	mean ± SD
Baseline Uric acid level (mg/dL)	6.07(±1.6)	5.7(±1.6)	5.35(±1.3)	5.41(±1.3)
BMI	27.37(±4.1)	28.31(±4.9)	27.13(±4.6)	26.95 (±4.44)
Baseline MDS-UPDRS III	4.6(±3.9)	2.72(±3.4)	20.4 (±8.75)	1.2 (±2.2)
Baseline MoCA	25.5 (±4.13)	27.3 (±1.71)	27.14 (±2.32)	28.2 (±1.1)

MDS-UPDRS, Movement Disorder Society-Unified Parkinson’s Disease Rating Scale; MoCA, Montreal Cognitive Assessment test; BMI, body mass index; SD, standard deviation.

Furthermore, longitudinal (Between subjects effects, *p* = 0.008) but not baseline serum uric acid measurements (*p* = 0.236) were higher in the Hyposmic cohort as compared to the PD cohort. [Baseline Hyposmic subgroup uric acid level was 5.7 mg/dL (95% CI 5.08 to 6.32) vs. Baseline PD 5.35 mg/dL (95% CI 5.23 to 5.47)].

There was no significant effect of Time or Time*Group interaction on uric acid level (Within subject effects, *p* = 0.164 and *p* = 0.571). Finally, baseline and longitudinal serum uric acid measurements did not differ statistically between Hyposmic subjects and healthy controls (despite a trend for higher uric acid in the Hyposmic group) (*p* = 0.896 and *p* = 0.327) and no significant effect of Time or Time*Group interaction on uric acid level was evidenced (Within subject effects, *p* = 0.745 and *p* = 0.240) (Fig. 1B). [Baseline Hyposmic subgroup uric acid level was 5.7 mg/dL (95% CI 5.08 to 6.32) vs.vs. Baseline HC 5.41 mg/dL (95% CI 5.23 to 5.59)].

No difference in serum uric acid levels was found between hyposmic patients who converted to PD compared to those who did not convert (5.89 mg/dL vs. 5.61 mg/dL, *p* = 0.699). On the other hand, patients in the RBD subgroup who converted to PD had a trend for lower uric acid levels compared to those who did not convert (5.56 mg/dL vs. 6.33 mg/dL), although this difference failed to reach significance (*p* = 0.163).

RBD non converters had significantly higher baseline serum uric acid as compared to HC (6.33 mg/dL vs. 5.41 mg/dL, *p* = 0.002), while there was no difference between Hyposmic non converters and HC (5.61 mg/dL vs. 5.41 mg/dL, *p* = 0.548).

No difference could be evidenced in baseline serum uric acid levels between RBD and Hyposmia participants mentioned above (6.07 and 5.7) and the additional group of 57 RBD and 93 Hyposmic individuals who were excluded from follow up in PPMI due to a normal DATSCAN (5.98 and 5.65 respectively).

In the RBD group, we observed a positive correlation between the duration of RBD (time since first reported RBD symptoms) and the increases in MDS-UPDRS score part III scores between baseline and year 2 [Spearman correlation: *r* = 0.467 (*p* = 0.004)] and between baseline and year 4 [Spearman correlation: *r* = 0.381 (*p* = 0.024)]. There was no significant correlation between baseline uric acid level or 2-year and 4-year change in uric acid and the duration of RBD symptoms.

Furthermore, there was no significant correlation between baseline uric acid level or 2-year and 4-year change in uric acid and either yearly MDS-UPDRS III scores (bl to year 5) or the increases in MDS-UPDRS score part III scores between baseline-year 2 and between baseline-year 4. No other parameter including MoCA score correlated to baseline uric acid level in a statistically significant way. Finally, we failed to reveal any significant impact of initial uric acid levels on the severity of motor deterioration when comparing high and low uric acid subgroups. It appears that RBD participants with high or low levels of uric acid in the initial measurement did not differ statistically regarding UPDRS III score during either the screening visit or V12 visit (year 5) (*p* = 0.255 and *p* = 0.54 respectively). The same was true for the Hyposmic cohort: participants with high or low levels of uric acid in the initial measurement did not differ statistically regarding UPDRS III score during either the screening visit or V12 visit (year 5) (*p* = 0.07 and *p* = 0.63 respectively).

## DISCUSSION

The role of blood uric acid as a biomarker in symptomatic motor PD has been increasingly established in the literature. Our present report assessed the role of serum uric acid as a putative biomarker in a prodromal PD cohort followed longitudinally. The RBD subgroup participants exhibited increased serum uric acid level as compared to idiopathic PD both at baseline and during a 5-year follow up, and this was also true for the hyposmic subgroups regarding follow up.

These data indicate that the well-established lowering of serum uric acid observed in PD occurs with the transition to clinical motor PD, and furthermore hint to the possibility that higher levels of serum uric acid in prodromal PD, due to inherent antioxidant properties, may provide protection against conversion to full-blown PD. According to this notion, the PPMI prodromal subjects studied here, with evidence of dopaminergic degeneration as exemplified by abnormal DATSCAN, might not have progressed to manifest PD due to an adequate endogenous antioxidant defense system which may prevent or delay further neurodegeneration. This is just one interpretation of our results, and alternatives include that something about the process of PD might cause low uric acid (reverse causation) or that low uric acid is a biomarker of some other more relevant mechanism in manifest PD [12]. Regarding reverse causation various hypotheses that may explain uric acid decrease as a consequence of established PD have been proposed. Alterations in gut microbiota reported in PD patients may also influence uric acid levels and therefore represent a putative confounder [13]. Moreover, in a large hospital database study, a diagnosis of PD was associated with a significant decrease in the subsequent risk of gout, implying that decreased uric acid levels are a feature of PD rather than a protective factor [14].

Two additional aspects of our results are also worth considering, although, due to the low numbers, they present just trends without statistical significance. In both prodromal subgroups levels of uric acid were consistently higher than controls, especially at earlier time points (Fig. 1A, B), This hints to the possibility that there may even be a compensatory increase in uric acid levels early on in the PD process, that is then reversed at later time points of disease progression. This idea is reinforced by the fact that RBD non-converters actually did show a statistically significant increase of uric acid levels compared to HCs. We also observed a trend for lower uric acid amongst RBD participants who phenoconverted compared to those who did not, supporting again the idea of a link between the emergence of PD motor symptoms and lowering of uric acid levels. At this point we have to highlight the fact that the number of prodromal patients who phenoconverted to PD during this follow-up period was rather low (13/39 of RBD and 8/26 of hyposmic group), therefore the lack of statistical significance is expected.

Literature data on uric acid level in prodromal PD are sparse. In a large Chinese study with more than 12,000 individuals followed longitudinally, higher average uric acid concentrations were associated with a lower likelihood of having possible RBD (but not new-onset possible RBD). The observed association appeared to be unrelated to potential risk factors for a vascular cause, such as BMI and hypertension, as well as other putative confounders. However, the authors admit that confounders might account for such results due to the observational nature of the study [15]. As far as prodromal PD is concerned, large retrospective and prospective studies have demonstrated decreased serum uric acid in individuals who eventually developed motor PD during follow up [16]. High serum uric acid levels have been correlated with lower risk of PD, a milder disease course and with the more benign tremor dominant motor subtype [17, 18]. As far as cognitive outcomes are concerned, lower levels of serum uric acid in the early disease stages were reported to be associated with the later occurrence of mild cognitive impairment (MCI) in an early PD cohort [19]. However, the causal relationship between high plasma urate and low risk of PD and its putative prognostic value have been questioned by other researchers [20, 21].

In accordance with our results, Uribe-San Martín and co-authors evaluated plasma uric acid levels in 24 patients with RBD and their association to PD. Patients were divided into 2 groups according to the presence or absence of PD, and it appeared that patients without PD and those who had more than 5 years of RBD exhibited higher levels of uric acid than patients with PD. The authors concluded that higher levels of plasma uric acid were associated with a longer duration of RBD without converting to PD, exhibiting resistance to the development of motor symptoms [22]. Moreover, in patients without PD, there was a positive correlation between years of evolution of RBD and the levels of uric acid. However, in our study, no such correlation between level of uric acid and duration of RBD symptomatology could be established in the RBD subgroup.

The notion of uric acid homeostasis contribution to the resistance of prodromal patients to PD could also apply to non manifesting carriers of pathogenic PD mutations. Bakshi and co-authors [23] evaluated the putative role of uric acid as a candidate biomarker of PD risk modulation in pathogenic LRRK2 mutation carriers using data from the LRRK2 Cohort Consortium and the PPMI. According to their observations, non manifesting LRRK2 mutation carriers had significantly higher levels of uric acid compared to those who developed PD and this applied to both sexes. The authors reached the conclusion that uric acid monitoring could be used as a biomarker of resistance to PD among LRRK2 mutation carriers [23].

Regarding the association of uric acid level in prodromal PD with motor or cognitive outcomes, no firm correlations could be established in our report. Past studies had contradictory outcomes regarding this issue. In an observational study, no clear relation between serum uric acid levels and motor scores could be evidenced. Moreover, such levels were negatively associated to non-motor symptoms burden in PD patients with a specific link to sleep/fatigue and cardiovascular domains [24]. A systematic review study evaluated the influence of serum uric acid level on non-motor symptoms occurrence and severity in patients with idiopathic PD, including attention/memory [25]. The authors failed to report any definite correlation between serum uric acid level and the occurrence and worsening of non-motor symptoms in PD. In contrast, another previous study assessed how levels of serum uric acid affect cognition in patients with RBD. The low uric acid group had worse scores in various aspects of cognitive function, including memory, executive function, and language, compared to healthy controls, whereas the scores of the high urate group were either intermediate, or similar to healthy controls [26]. In another study during the motor PD phase, uric acid and uric acid/creatinine levels in the early and medium stage PD patients were significantly higher than in the advanced stage ones [27, 28]. Accordingly, if we consider prodromal and motor PD to be the two edges in a continuum, higher uric acid could possibly mark the initial phases of the disorder and a gradual decrease of uric acid level might simply reflect disease progression and severity.

An important merit of our report is that data we used from the PPMI database have been collected and processed uniformly across PPMI centers, with a thorough standardized clinical and laboratory assessment over a prolonged period of time. On the other hand, a major limitation is the relatively small number of RBD and Hyposmia subjects. We should also highlight the possibility that with this data set at 5-year time interval, and with this small number of patients, it is difficult to comment on the temporal nature of uric acid over the disease process. Finally, it appears that RBD and Hyposmic group have a larger standard deviation (SD) concerning uric acid levels than PD and HC. This could be attributed to the relatively small size of these subgroups and/or to the heterogeneity in thesepopulations.

Regarding implications for therapeutic interventions, since a clinical study with inosine, a uric acid-boosting compound, has failed to demonstrate a protective effect in established motor PD [29, 30], a possibility to consider for future trials, given our results, is to examine such compounds in late prodromal stages just before phenoconversion, if this time point can be well defined.

## Supplementary Material

Supplementary Material

## Data Availability

The data supporting the findings of this study are available on request from the corresponding author. The data are not publicly available due to privacy or ethical restrictions.
